# Phloem-Conducting Cells in Haustoria of the Root-Parasitic Plant *Phelipanche aegyptiaca* Retain Nuclei and Are Not Mature Sieve Elements

**DOI:** 10.3390/plants6040060

**Published:** 2017-12-05

**Authors:** Minako Ekawa, Koh Aoki

**Affiliations:** Graduate School of Life and Environmental Sciences, Osaka Prefecture University, 1-1 Gakuen-Cho, Naka-Ku, Sakai, Osaka 599-8531, Japan; swc02013@edu.osakafu-u.ac.jp

**Keywords:** haustorium, *NAC45*, *NEN1*, *NEN4*, *Phelipanche aegyptiaca*, phloem, sieve element

## Abstract

*Phelipanche aegyptiaca* parasitizes a wide range of plants, including important crops, and causes serious damage to their production. *P. aegyptiaca* develops a specialized intrusive organ called a haustorium that establishes connections to the host’s xylem and phloem. In parallel with the development of xylem vessels, the differentiation of phloem-conducting cells has been demonstrated by the translocation of symplasmic tracers from the host to the parasite. However, it is unclear yet whether haustorial phloem-conducting cells are sieve elements. In this study, we identified phloem-conducting cells in haustoria by the host-to-parasite translocation of green fluorescent protein (GFP) from *AtSUC2pro::GFP* tomato sieve tubes. Haustorial GFP-conducting cells contained nuclei but not callose-rich sieve plates, indicating that phloem-conducting cells in haustoria differ from conventional sieve elements. To ascertain why the nuclei were not degenerated, expression of the *P. aegyptiaca* homologs NAC-domain containing transcription factor (*NAC45*), NAC45/86-dependent exonuclease-domain protein 1 (*NEN1*), and *NEN4* was examined. However, these genes were more highly expressed in the haustorium than in tubercle protrusion, implying that nuclear degradation in haustoria may not be exclusively controlled by the *NAC45*/*86*-*NEN* regulatory pathway. Our results also suggest that the formation of plasmodesmata with large size exclusion limits is independent of nuclear degradation and callose deposition.

## 1. Introduction

Parasitic plants belonging to the genera *Orobanche* and *Phelipanche* are obligate parasites that subsist on hosts’ roots. *Phelipanche aegyptiaca* parasitizes a wide range of plants, including important crops such as tomato, melon, and legumes, and causes serious damage to crop production [1]. *P. aegyptiaca* develops a radicle upon germination when perceiving a stimulant exuded from the hosts’ roots. When the radicle contacts the host root, a haustorium (a specialized intrusive organ) differentiates from the terminus of the radicle and reaches the host’s vascular tissues, and the vascular conducting tissues then differentiate. After establishing the conducting tissues, *P. aegyptiaca* initiates the development of a tubercle, which is a storage organ developed from the seedling remaining outside the host’s root [2]. Formation of vascular conducting tissue in haustoria is necessary for obligate parasitic plants to absorb water and nutrients from the hosts, because they lack roots or photosynthetic organs.

Xylem and phloem differentiation in haustoria are pivotal steps in the successful establishment of a parasitic connection. Xylem differentiation is characterized by the formation of vessel elements with highly lignified cell walls [3]. Elongated cells in the haustorial tips of *Striga hermonthica* perforate cell walls of the host’s xylem vessel, and the invading part of the contact cell (the osculum) loses its cytoplasmic contents and differentiates into a water-conducting vessel element [3]. In a *P. aegyptiaca*-*Arabidopsis thaliana* parasitic complex, exogenous auxin application decreases the infection rate, suggesting that auxin flow plays an important role in xylem continuity [4]. Open xylem connections at the parasite-host interface allow a flow of water and minerals to the parasitic plant.

Haustorial phloem differentiation has also been described. Studies on the haustiorial phloem of various parasitic plants showed that the presence of sieve elements (SEs) in haustoria depends upon the species in question. A morphological study using light microscopy on the structure of the haustorium of a stem parasitic plant, *Cuscuta gronovii*, has demonstrated that most of the cells in sieve tubes have prominent nuclei and sieve plates, and no sieve-tube members reach the host phloem [5]. In contrast, morphological studies of haustoria of a stem obligate parasitic plant, *Cuscuta odorata*, using electron microscopy have demonstrated that there are sieve element (SE)-companion cell (CC) complexes in haustoria that are directly in contact to host SEs [6]. A root facultative parasitic plant in the *Orobancaceae* family, *Lathraea*, does not develop phloem in haustoria, and it takes organic substances through xylem. Another root facultative parasitic plant, *Alectra vogelii*, develops SEs in haustoria, but they do not have a direct contact to the host’s SE [6]. In secondary haustoria of a root obligate parasitic plant, *Phelipanche ramosa*, SEs appear to develop, however, a final cell that attaches to host’s SE is of parenchymatous nature [6]. On the other hand, transport function of haustorial phloem has been demonstrated by using symplasmic tracers. Studies using fluorescent symplasmic tracers have demonstrated that carboxyfluorescein or CC-expressed green fluorescent protein (GFP) can be transported from host to parasite through haustoria in *Cuscuta reflexa* [7,8], *Phelipanche ramosa* [9], and *P. aegyptiaca* [10]. In haustoria of a facultative parasitic plant, *Phtheirospermum japonicum*, carboxyfluorescein was transported from the host’s sieve tubes to haustoria, but CC-expressed GFP was not, implying the lack of phloem-to-phloem connection between the host and parasite, or difficulty in GFP movement due to its size [11]. Despite the exception of *Lathraea* and *P. japonicum*, these studies on the cell morphology and the transport function collectively suggested that it is likely that phloem contents should be transported from the host to parasitic plants by haustorial phloem. However, because few previous studies examined the cell morphology and transport function of the haustorial phloem simultaneously, it is still unclear whether the pathway in which phloem contents are transported occurs in SEs.

In this study, we observed the transport function of phloem-conducting cells in haustoria and their morphology at the same time. We identified phloem-conducting cells in haustoria by the plant-to-plant translocation of GFP from *AtSUC2pro::GFP* tomato sieve tubes. GFP-conducting cells contained nuclei but not callose-rich sieve plates, indicating that phloem-conducting cells in haustoria differ from conventional sieve elements (SE). However, normal SEs were present in phloem-conducting cells in tubercle protrusions. The retention of nuclei and lack of sieve plates suggest that SE maturation is retarded in the haustoria. We also investigated the expression profiles of genes involved in SE differentiation, including homologs of *NAC-DOMAIN CONTAINING TRANSCRIPTION FACTOR* (*NAC45*), *NAC45*/*86-DEPENDENT EXONUCLEASE-DOMAIN PROTEIN 1* (*NEN1*), and *NEN4*. The relative expression levels of these genes were higher in haustoria than in the tubercle protrusions. This result was not consistent with the immaturity of sieve elements; therefore, nuclear degradation in haustorial GFP-conducting cells may not be controlled exclusively by the *NAC45*/*86*-*NEN* regulatory pathway. Our results also suggest that formation of plasmodesmata with a large size exclusion limit (SEL) is independent of nuclear degradation and callose deposition.

## 2. Results

### 2.1. GFP Expressed in Phloem of Host Plant Visualized a Symplasmic Pathway of a Parasitic Plant

**Figure 1:**
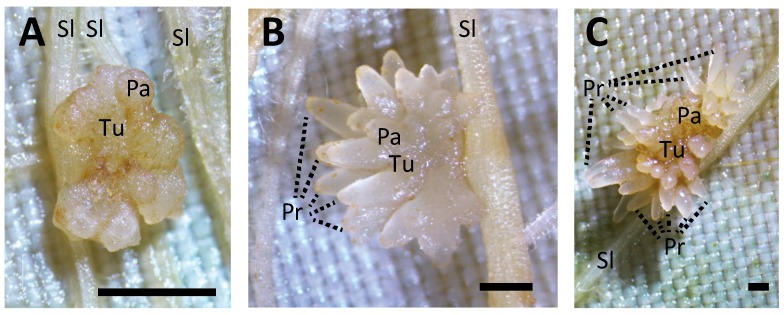
Appearance of *Phelipanche aegyptiaca* tubercles at different parasitic stages. The tubercle diameters are (**A**) 1.5 mm, (**B**) 3 mm, and (**C**) 1 cm. Scale bar: 1 mm. Tu, tubercle; Pr, protrusion; Sl, tomato; Pa, *P. aegyptiaca*.

**Figure 2:**
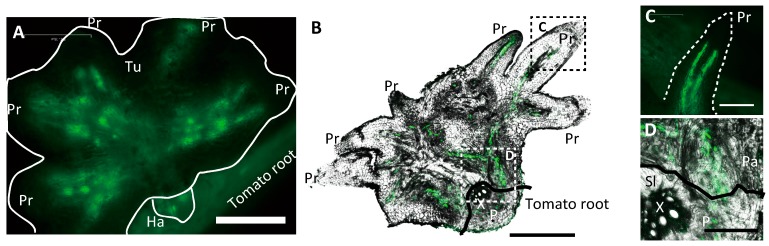
Translocation of green fluorescent protein (GFP, green) from *AtSUC2::GFP* tomato roots to a tubercle. (**A**) GFP in a 3-mm-diameter tubercle. Scale bar: 500 μm. (**B**) GFP detected by immunostaining in a paraffin section of a 3-mm-diameter tubercle. Scale bar: 250 μm. (**C**) Magnified image of the rectangle C in Panel (B). GFP in a protrusion. Scale bar: 100 μm. (**D**) Magnified image of the rectangle D in Panel (B). GFP in the tomato-*Phelipanche aegyptiaca* interface. Scale bar: 100 μm. Tu, tubercle; Ha, haustorium; Pr, protrusion; X, xylem of tomato; P, phloem of tomato; Sl, tomato; Pa, *P. aegyptiaca*.

We traced a symplasmic pathway between *AtSUC2pro::GFP* tomato and *P. aegyptiaca*. The developmental stages of the *P. aegyptiaca* tubercles were judged by their diameters, which were 1.5 mm, 3 mm, and 1 cm (Figure 1A–C). In the 3-mm-diameter tubercle, GFP was clearly detected in the haustoria and tubercle protrusions (Figure 2A), so it was transported a long distance from host sieve tube to the haustoria and protrusions through the symplasmic pathway. To visualize the cell architecture more clearly, we made paraffin sections and detected GFP by immunostaining (Figure 2B). In the protrusions, GFP was detected in two poles in the stele (Figure 2C), suggesting that in protrusion, phloem-conducting cells are spatially arranged in a similar manner to that in the roots of vascular plants. In contrast, in haustoria, GFP-conducting cells appeared to be arranged more irregularly (Figure 2D).

### 2.2. Nuclei Were Present in GFP-Conducting Cells

**Figure 3:**
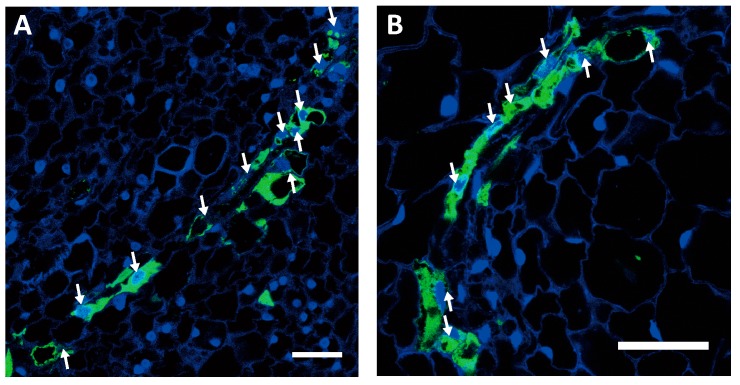
Green fluorescent protein (GFP)-conducting cells containing nuclei in the haustoria of (**A**) a 1.5-mm-diameter tubercle and (**B**) a 3-mm-diameter tubercle. White arrows indicate nuclei. Most of the GFP-conducting cells contained nuclei. Scale bar: 50 μm.

Most of the GFP-conducting cells in the haustoria contained nuclei. We stained haustorial sections from the 1.5-mm-diameter and 3-mm-diameter tubercles with 4′6-diamidino-2-phenylindole (DAPI) (Figure 3A,B), and we found that GFP-conducting cells in haustoria of both the 1.5-mm-diameter tubercle, which did not seem to have any protrusions, and the 3-mm-diameter tubercle, which had elongated protrusions, contained nuclei. This suggests that GFP-conducting cells in the haustoria do not develop into mature SEs.

### 2.3. GFP-Conducting Cells in Haustoria Were Not Mature SEs

**Figure 4:**
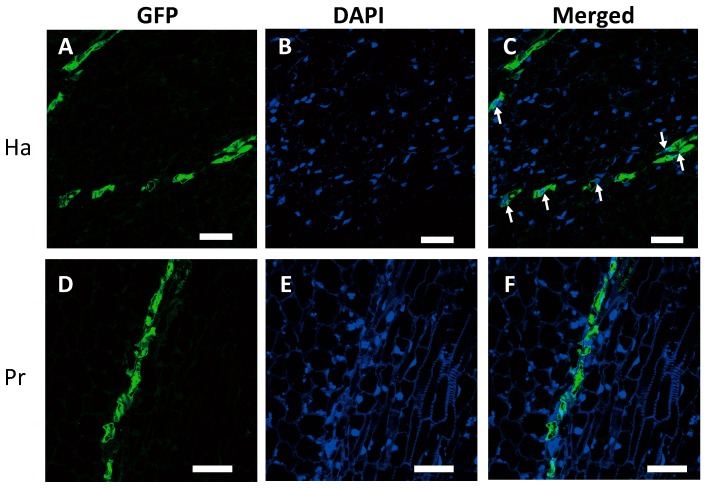

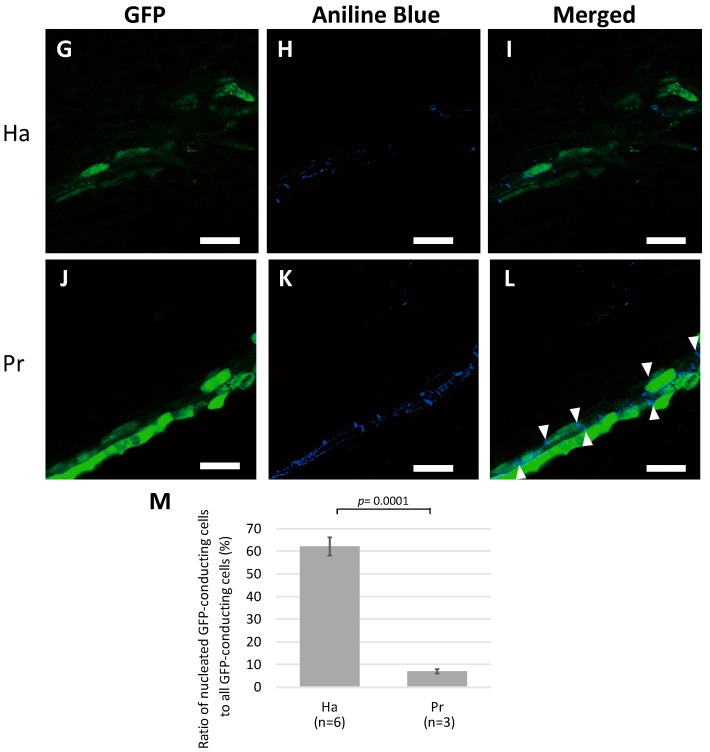
Detection of nuclei and callose-rich sieve plates in green fluorescent protein (GFP)-conducting cells in a 1-cm-diameter tubercle. (**A**–**C**) 4′6-diamidino-2-phenylindole (DAPI) staining of haustorial cells; (**A**) GFP, (**B**) DAPI, and (**C**) merged. (**D**–**F**) DAPI staining of protrusion cells; (**D**) GFP, (**E**) DAPI, and (**F**) merged. White arrows indicate nuclei. (**G**–**I**) Aniline Blue staining of haustorial cells; (**G**) GFP, (**H**) Aniline Blue, and (**I**) merged. (**J**–**L**) Aniline Blue staining of protrusion cells; (**J**) GFP, (**H**) Aniline Blue, and (**I**) merged. White triangles indicate sieve plates. Scale bar: 50 μm. (**M**) Percentage of nucleated GFP-conducting cells to all GFP-conducting cells. Ha, haustorium; Pr, protrusion. The means and standard deviations of six and three different haustorial and protrusion specimens, respectively, are presented. *n*: number of specimens.

To ascertain whether GFP-conducting cells in haustoria are SEs, we detected nuclei with DAPI staining and callose-rich sieve plate with Aniline Blue staining using the developed, 1-cm-diameter tubercle. In the haustoria, nuclei were co-localized with GFP-conducting cells (Figure 4A–C); however, callose deposition was not detected in cell walls between adjacent GFP-conducting cells, indicating that no sieve plates had developed (Figure 4G–I). However, in the protrusions, few nuclei were found in GFP-conducting cells (Figure 4D–F), whereas callose deposition was clearly detected between neighboring GFP-conducting cells, indicating the presence of sieve plates (Figure 4J–L). To confirm that the nuclei were retained, we counted the number of GFP-conducting cells with or without nuclei in the haustorial and protrusion sections (Figure 4M). In the haustoria, nuclei were detected in approximately 62% of GFP-conducting cells. In the protrusions, nuclei were detected in approximately 7% of GFP-conducting cells, which was a significantly lower percentage than that in the haustoria. This result demonstrates that GFP-conducting cells in haustoria are not mature SEs, whereas those in protrusions are.

### 2.4. SE Differentiation-Related Genes Were Expressed in Haustoria

**Figure 5:**
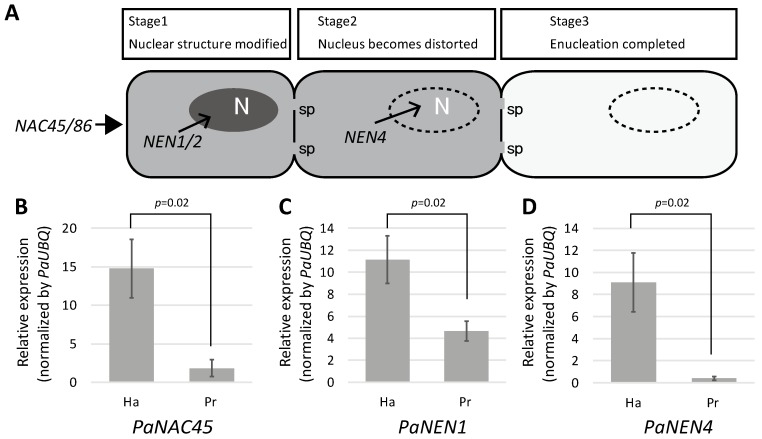
(**A**) Stages of sieve element differentiation. N, nucleus; sp, sieve plate pore. (**B**–**D**) Relative expression levels of (**B**) *PaNAC45*, (**C**) *PaNEN1*, and (**D**) *PaNEN4* in a surgically separated haustorium (Ha) and protrusion (Pr). Expression levels were normalized to that of *Phelipanche aegyptiaca UBIQUITIN* (*PaUBQ*). The means and standard deviations of three replicates are presented. NAC = NAC-domain containing transcription factor; NEN = NAC45/86-dependent exonuclease-domain protein.

SE differentiation is regulated by the transcription factor NAC45/86 [12]. NAC45/86 positively controls the expression of *NEN1* to *NEN4*, which are responsible for the degradation of nuclei in SE precursor cells (Figure 5A). Nuclear retention in haustorial GFP-conducting cells may have been caused by the suppression of *NAC45/86* and *NEN* expression to lower than that in the protrusions, which resulted in the inhibition of nuclear degradation in the haustoria. To test this hypothesis, we dissected a tomato root containing the haustorium and the rest of the tubercle (diameter 3 ± 1 mm), and quantified the relative expression levels of *PaNAC45*, *PaNEN1*, and *PaNEN4*. Unexpectedly, the relative expression levels of all three genes were significantly higher in the haustorium than in the protrusion (Figure 5B–D), suggesting that SE differentiation is indeed activated in the haustorium.

## 3. Discussion

### 3.1. Haustorial Phloem-Conducting Cells Are Not Mature SEs

The movement of GFP that is expressed in the host’s CCs to parasitic plants has been reported in a stem parasitic plant (*Cuscuta reflexa*) [7,8] and root parasitic plants (*P. ramosa* and *P. aegyptiaca*) [9,10]. GFP is smaller (27 kDa) than the typical size exclusion limit (SEL) of plasmodesmata between CCs and SEs (that can be as large as 67 kDa) [13], so GFP synthesized in CCs can be translocated to SEs. In addition, in *Arabidopsis* and tobacco plants that express *AtSUC2pro::GFP*, GFP is restricted to the phloem [14]. These results suggest that GFP expressed in CCs is translocated within the SE-CC complex, and the translocation of GFP into parasitic plants means that there should be a continuity of sieve tubes across the boundary between the host and parasitic plants, and GFP moves non-selectively from the source (host) to the sink (parasite) following the phloem translocation stream.

GFP-conducting cells in the haustoria of *P. aegyptiaca* contained nuclei but not sieve plates (Figure 3A,B and Figure 4C). Conventional sieve tube elements are characterized by the elimination of cellular components, including nuclei during development [15] and callose deposition in sieve plates [16]. The apparent lack of these attributes first led us to examine whether *P. aegyptiaca* is capable of developing SEs in other organs. In the tubercles, GFP moved a long distance to the protrusions, and GFP-conducting cells were clearly visible there (Figure 2C). In contrast to in haustoria, GFP-conducting cells in the protrusions had sieve plates, and fewer nuclei than the surrounding cells (Figure 4J–L). Therefore, *P. aegyptiaca* should not have any defects in the development of SEs.

The presence of nucleated SEs in haustoria have been reported in *C. gronovii* [5]. In *C. gronovii*, it has been reported that most of the cells in haustorial sieve tubes, which were recognized as continuous strands of elongated cells which made connection with the host phloem, had prominent nuclei [5]. The nucleated phloem-conducting cells of *P. aegyptiaca* are similar to SEs of *C. gronovii*. However, the study using *C. gronovii* did not clarify whether phloem transport occurred in “the continuous strands of elongated cells”. In this study, we clearly identified phloem-conducting cells in *P. aegyptiaca* haustoria by tracing the transport of contents of host’s sieve tubes (i.e., GFP), and demonstrated that the GFP-conducting cells were nucleated and lacked sieve plates (Figure 3 and Figure 4).

To explain the retention of nuclei, we first hypothesized that SE differentiation activity could be low in haustoria, and, consequently, SE maturation is retarded. However, the expression level of *PaNAC45*, which is responsible for the regulation of SE differentiation, and the expression levels of *PaNEN1* and *PaNEN4*, which are responsible for nuclear degeneration [12], were higher in the haustoria than in the protrusions (Figure 5B–D), which does not support the hypothesis that SE differentiation activity is low in haustoria.

### 3.2. Why Do Haustorial Phloem-Conducting Cells Fail to Undergo Nuclear Degradation?

It has recently been reported that nucleated SEs were present in an *Arabidopsis* loss-of-function mutant of genes similar to *SUPPRESSOR OF MAX2 1-LIKE* (*SMXL*), *smxl4;smxl5* [17]. Although functions of *SMXL4* and *SMXL5* were finally shown to be independent of strigolactone and karrikin signaling [17], they were shown to be involved in differentiation of protophloem in the root, since in cells located at positions expected for SEs, degradation of nuclei did not occur [17]. However, expression levels of genes involved in SE differentiation, including *NEN4*, *NAC86* [12], *ALTERED PHLOEM DEVELOPMENT* (*APL*) [18], and *CALLOSE SYNTHASE 7* (*CALS7*) [19], were lower than those of wild type *Arabidopsis* during induction of phloem differentiation using VISUAL [20], which is different from the gene expression profiles observed in haustoria of *P. aegyptiaca* (Figure 5B–D). *SMXL4* and *SMXL5* might regulate the *NAC*-*NEN* regulatory pathway of phloem differentiation [21]. We thus speculate that retention of nuclei in haustorial GFP-conducting cells may not be due to the repression of *SMXLs*.

Retention of nuclei in the root protophloem was also observed in a triple mutant of brassinosteroid (BR) receptor genes, *brassinosteroid insensitive 1* (*bri1*); *bri1-like 1* (*brl1*); *bri1-like 3* (*brl3*) [22]. The BR signaling pathway is known to be activated by a polarly localized membrane-associated protein OCTOPUS (OPS) [23]. Morphologies of SEs in *bri1*;*brl1*;*brl3* and *ops* mutants were similar to that of haustorial GFP-conducting cells. In roots of *bri1*;*brl1*;*brl3* and *ops* mutants, differentiation of SEs in the protophloem cell file was discontinuous, and nuclear degradation or callose deposition were not seen in the protophloem cell file gaps, indicating that loss-of-function of BR receptor genes and *OPS* delays protophloem differentiation [22,24]. A loss-of-function mutant of a homolog of *OPS*, *OCTOPUS-LIKE 2* (*OPL2*), also had a defect in metaphloem differentiation [25]. It has been proposed that the *NAC*-*NEN* regulatory pathway is not directly coupled to the BR signaling pathway including *OPS*, although a crosstalk between these pathways has not been studied in detail [21]. If repression of *OPS* does not down-regulate *NEN* genes, expression levels of *NEN* genes could stay high in differentiating SEs, and SEs possibly retain nuclei like in the *ops* mutant. This argument tempts us to hypothesize that nuclear retention in haustorial GFP-conducting cells may be due to the repression in the BR-perception associated with the downregulation of an *OPS* ortholog of *P. aegyptiaca*. Identification of *P. aegyptiaca OPS* is currently underway.

A hypothesis from a different point of view for the nuclear retention is that exonuclease activities of NEN1 and NEN4 may be repressed by an inhibitor protein. In the case of apoptosis, a major apoptotic nuclease, Caspase-activated DNase (CAD), is deactivated by complex formation with the inhibitor of CAD (ICAD) [26]. During apoptotic execution of nuclear degradation, ICAD is cleaved by a protease, caspase, and then CAD is liberated to form an active homo-dimer [27]. We speculate that NEN1 and NEN4 might be deactivated in haustorial GFP-conducting cells by a nuclease inhibitor, although the inhibitor needs to be identified.

### 3.3. Retention of Nuclei Is Independent of Other SE Differentiation Processes

In the haustorial GFP-conducting cells, the nuclei were retained not only in developing tubercles that were 1.5 mm and 3 mm in diameter (Figure 3A,B), but also in developed, 1-cm-diameter tubercles (Figure 4A–C). This suggests that nuclear retention in phloem-conducting cells occur in haustoria in an organ-specific manner. Our findings clearly demonstrate that phloem-conducting cells retain their nuclei and do not develop sieve plates even after an array of cells has started transporting phloem materials, suggesting that nuclear degradation and sieve plate formation are coordinated, while the formation of plasmodesmata with large SEL is independent of them. It has been suggested that degradation of nucleus controlled by the *NAC45*-dependent regulatory pathway is independent of the callose accumulation [12]. Regarding the formation of plasmodesmata with large SEL, it has been reported that development of the sieve plate pores is affected by CHOLINE TRANSPORTER-LIKE 1 (CHER1), since *cher1-1* mutant of *Arabidopsis* has been shown to have reduced phloem conductivity [28]. It is currently unclear whether *CHER1* is controlled by the same upstream transcriptional regulator as those controlling the nuclear degradation or callose deposition. Taken together, it can be hypothesized that the processes of sieve element differentiation, such as nuclear degeneration, sieve plate formation, and formation of plasmodesmata with large SELs, may be controlled independently. Regulatory relationships between the pathways that lead to these cellular events remain to be investigated.

## 4. Materials and Methods

### 4.1. Plant Materials

*P. aegyptiaca* seeds were stored at 4 °C in the dark. The seeds were surface-sterilized with 1 mL of 99.5% (*v/v*) ethanol for 1 min and 1 mL of 1% (*v/v*) sodium hypochlorite (Wako Pure Chemical Industries, Ltd., Osaka, Japan) for 10 min, twice. After washing with sterile distilled water three times, the seeds were spread on sterilized glass filter paper (ADVANTEC, Tokyo, Japan) with 3 mL of sterile distilled water inside a plastic dish and incubated in the dark at 25 °C for 3 days. One day after applying the synthetic germination stimulant rac-GR24 (Chiralix, Nijmegen, Netherlands) (final concentration, 2 mg/L) to induce germination, the seeds on the glass filter paper were placed on Murashige and Skoog (MS) solid medium supplemented with MS Plant Salt Mixture (Wako Pure Chemical Industries, Ltd.), 3% (*w/v*) of sucrose (Wako Pure Chemical Industries, Ltd.), MS modified vitamin solution (1000×) (final concentration, 1×) (MP Biomedicals, Santa Ana, CA, USA), and 0.8% (*w/v*) of agarose with the pH adjusted to 5.8. The paper was then removed and the seeds were incubated in the dark at 25 °C. The host plants, *Solanum lycopersicum* ‘Micro-Tom’ (accession number TOMJPF00001, National Bioresource Project, Japan) and transgenic tomato plants (*Solanum lycopersicum* ‘M82’) expressing *AtSUC2pro::GFP*, were grown on rhizotrons under a 16/8 h light/dark cycle at 25 °C. Three days after the GR24 treatment, germinated *P. aegyptiaca* seeds were inoculated to 3-week-old host plants in rhizotrons and grown under a 16/8 h light/dark cycle at 25 °C.

### 4.2. Cloning Complementary DNA (cDNA) of PaNEN1, PaNEN4, and PaNAC45

To select *P. aegyptiaca* target genes, contigs of the Parasitic Plant Genome Project OrAeBC5 dataset (http://ppgp.huck.psu.edu/) were compared with the protein sequence of *Sesamum indicum* provided in Sinbase (http://ocri-genomics.org/Sinbase/) [29] using BLASTx [30]. Based on the top hits, OrAeBC5_5123, OrAeBC5_14368.1, and OrAeBC5_32545.1 were chosen for *PaNEN1*, *PaNEN4*, and *PaNAC45*, respectively. For cloning, gene-specific primers were designed based on the sequence of each contig (Table 1). For cDNA isolation, *P. aegyptiaca* tubercles that were frozen in liquid nitrogen were homogenized using a pestle and mortar. Total RNA was extracted using a Qiagen RNeasy Plant Mini Kit (Qiagen, Hilden, Germany), according to the manufacturer’s protocol. cDNA was synthesized using a ReverTra Ace^®^ quantitative polymerase chain reaction (qPCR) reverse transcription (RT) kit (Toyobo, Osaka, Japan) and an oligo (dT) primer (Toyobo). Using gene-specific primers, partial sequences of the target genes were amplified, extracted from agarose gel using a Wizard^®^ SV Gel and PCR Clean-Up System (Promega, Madison, WI, USA), and cloned into a pCR™-Blunt II TOPO^®^ vector using a Zero Blunt^®^ TOPO^®^ PCR Cloning Kit (Thermo Fisher Scientific, Waltham, MA, USA). The cDNA sequences of *PaNAC45*, *PaNEN1*, and *PaNEN4* are provided in Supplementary Table S1. The nucleotide sequences reported in this paper have been submitted to the DNA Data Bank of Japan under accession numbers; *PaNAC45*: LC333147, *PaNEN1*: LC333148, and *PaNEN4*: LC333149.

### 4.3. Real Time PCR

Two weeks after parasitization on the ‘Micro-Tom’ plant, parasitic interface tissues were surgically separated into ‘haustorium’ (Ha) and ‘protrusion’ (Pr) samples. RNA was extracted from each sample (approximately 100 mg) and treated with DNase using a TURBO DNA-free™ Kit (Thermo Fisher Scientific). cDNA was synthesized using a ReverTra Ace^®^ qPCR RT kit (Toyobo) and an oligo (dT) primer (Toyobo). Based on previous studies [31,32], the stable-expression gene *Ubiquitin1* (*PaUBQ1*) (OrAeBC4_32941) was chosen as a reference gene for normalization. In order to avoid amplification of the host’s genes, primers were designed based on untranslated region sequence of each contig and confirmed parasite-specific amplification. qRT-PCR was performed using SYBR^®^ Green on Applied Biosystems StepOne™ Real Time PCR System (Thermo Fisher Scientific). PCR reactions were performed in 20-μL total volume (per well) that contained 1 μL of cDNA, 0.4 μL each of the gene-specific primers (10 μM), 10 μL of Fast SYBR™ Green Master Mix (Thermo Fisher Scientific), and water, according to the manufacturer’s protocol. The annealing temperature was set at 56 °C. For generating standard curves of target genes, cDNA from *P. aegyptiaca* protrusion tissue was diluted to 1:5, 1:25, 1:125, and 1:625. Data were analyzed using StepOne^TM^ Software v2.3 (Thermo Fisher Scientific) and normalized to the *PaUBQ1* expression level.

### 4.4. Preparation and Imaging of Agarose-Embedded Sections

Two weeks after parasitization on *AtSUC2pro::GFP* tomato plants, parasitic interface tissues were cut and embedded in 5% (*w*/*v*) agarose without fixation, and cut using MicroSlicer^TM^ ZERO 1N (DOSAKA, Kyoto, Japan) into 100-μm sections. The sections were then stained with 1% (*w*/*v*) Aniline Blue (WALDECK GmbH & Co., KG, Waldeck, Germany) at room temperature for 2 h and washed with phosphate-buffered saline (PBS) (137 mM NaCl, 2.7 mM KCl, 8.1 mM Na_2_HPO_4_, and 1.47 mM KH_2_PO_4_) three times.

### 4.5. Immunostaining

Immunostaining was performed as previously described [33], with slight modifications. Two weeks after parasitization on *AtSUC2pro::GFP* tomato plants, parasitic interface tissues were fixed with 4% (*w/v*) paraformaldehyde phosphate buffer solution (Wako Pure Chemical Industries Ltd., Richmond, VA, USA) and embedded in paraffin (Paraplast, Leica Biosystems, Richmond, VA, USA) according to the conventional protocol [34]. Paraffin blocks were cut with a microtome (PR-50, Yamato Kohki, Saitama, Japan) into 10-μm sections and fixed on glass slides at 40 °C for 1 day. After deparaffinization, the sections were treated with blocking buffer (1% *w/v* bovine serum albumin (Nacalai Tesque, Kyoto, Japan) in PBS pH 7.0) for 2 h at room temperature in a humidified chamber and washed three times with PBS. They were then incubated with primary antibodies (monoclonal mouse anti-GFP antibody (Nacalai Tesque, Kyoto, Japan, 4363-24) 1:1000 in blocking buffer, and normal rat IgG 1:1000 in blocking buffer) over night at 4 °C in a humidified chamber. After washing three times with PBS, sections were incubated with secondary antibody (CF568 Donkey Anti-Mouse IgG (H + L) (Biotium, Fremont, CA, USA) that was highly cross-adsorbed, and diluted 1:750 in blocking buffer) for 2 h, washed three times with PBS, and incubated with 2 μg/mL DAPI (Nacalai Tesque) for 20 min at room temperature in a dark, humidified chamber. After removing excess buffer, the sections were encapsulated in SlowFade™ Diamond Antifade Mountant (Thermo Fisher Scientific).

### 4.6. Confocal Laser-Scanning Microscopy

Images were taken by using a confocal laser-scanning microscope (Leica 408 TCS SP8, Leica Microsystems, Wetzlar, Germany). GFP was excited at 488 nm and the light emitted was captured at 500 to 530 nm. CF568 was excited at 552 nm, and the light emitted was captured at 580 to 610 nm. DAPI and Aniline Blue were excited at 405 nm, and the light emitted was captured at 430 to 480 nm. Images were analyzed using Leica Application Suite X (Leica Microsystems).

## Figures and Tables

**Table 1:** Primers used in this study.

Primer ID	Sequence (5′ → 3′)
PaNEN1 forward	ACAAGCAAGCTATACATAACCGTG
PaNEN1 reverse	TAACAGCACCAAAGTTAAATCAAACC
PaNEN1 qPCR forward	ACAAGCAAGCTATACATAACCGTG
PaNEN1 qPCR reverse	TATTCAGGAAATAAAGAATAATGGGAGC
PaNEN4 forward	ACAGATGTAGTGTATGCTAGGATTACC
PaNEN4 reverse	ATAACGACACCATCTTACAAAGTTTGAAC
PaNEN4 qPCR forward	AGGAAGAACTCATTCTTGCTAACAAC
PaNEN4 qPCR reverse	TGACATTGTCAGTTGTAACATACTG
PaNAC45 forward	ATTTCAAAAAAGGACCAGGAGACTGG
PaNAC45 reverse	AGTTCATTGGCATTGGATATTCAGAAG
PaNAC45 qPCR forward	ATCAATGGCCGCAAAATCGATC
PaNAC45 qPCR reverse	TACCATTCAAGATCTTTGCTTGGC
PaUBQ1 qPCR forward	ATCACTGCCTGATTATCAGACGC
PaUBQ1 qPCR reverse	TAGGGAATCAACTCGATTATGGCG

## Supplementary Materials

The following are available online at www.mdpi.com/2223-7747/6/4/60/s1, Table S1: DNA sequences of *PaNEN1*, *PaNEN4,* and *PaNAC45* cDNAs.

## Abbreviations

BR	brassinosteroid
CC	companion cell
CHER1	CHOLINE TRANSPORTER-LIKE 1
DAPI	4′,6-diamidino-2-phenylindole
PBS	phosphate-buffered saline
qPCR	quantitative polymerase chain reaction
SE	sieve element
SEL	size exclusion limit
*SUC2*	*SUCROSE-PROTON SYMPORTER 2*
