# Supplementary material for: Phloem-Conducting Cells in Haustoria of the Root-Parasitic Plant Phelipanche aegyptiaca Retain Nuclei and Are Not Mature Sieve Elements
Source: Plants (Basel). 2017 Dec 5;6(4):60. doi: 10.3390/plants6040060 (PMC5750636; doi:10.3390/plants6040060)
Supplement: Supplementary file 1 [file plants-06-00060-s001.pdf]

Supplementary Table S1. DNA sequences of PaNEN1, PaNEN4 and PaNAC45 cDNAs.

>PaNEN1
ACAAGCAAGCTATACATAACCGTGAACTCTTTACATACACATATACTCACACACAGACACGGTGGGTA
GGTATTACATCTTCAAATTGGCTCCCATTCCTGAATAAACCTCTGAAACAAGAAAATGGCGGTTACAG
GCGACGATAAATCGGAGATAGCATTCTTCGACTTGGAGACGACTGTTCCGACCCGAACCGGTCAGGGT
CATGCCATTTTGGAATTCGGAGCCATTCTGGTTTGCCCCAAGAAGCTTGTGGAGCTTCGAAGTTACCCC
ACCTTGGTCCGACCCGCTGACCTCTCCCACATTACATCTCTTTCCGTCCGCTGCAATGGCATCACTAAA
GACGCCGTCGTTTCTGCACCTACTTTCGCTGATATTGCTGATATGGTCTACGAACTTCTTCACGGAAGA
ATATGGGCTGGTCATAATATATTGAGATTTGATTGTGCTCGTATAAGGGAGGCATATGCTGAGATTAA
CAAGCCAGTCCCGGAGCCTAAGGGAACTATTGATTCGCTAGCACTCTTAACACAAAAGTTTGGAAGGA
GAGCTGGTGATATGAAGATGGCATCTCTTGCAACATATTTTGGGCTTGGCCAGCAAACCCATAGGAGT
TTGGATGATGTTCGCATGAATCTGGAAGTCCTCAAGTACTGTGCAACTGTTCTATTTTTGGAATCTAGC
CTCCCAGACATATTTACGGAAAACAGTTGGGTTTCTCCGAACGCTACCACAAGAAGTCGAAGCAATGG
AAAAAGTTCTAGAGAGAAGAATGAAAGCACGCCATCTTCGAGTATCAACGTTGAATATCTTTCAACAT
CATCTCCACCTCATAACACAGTGGAGGAAAATCACCCTATACTGTCGCTTATGTCACCCAACACCGGT
GAAGTAAATTTGCCCCTCACTGAACCCAATCCAGCTGGAGCCGAACCTTTTAACATGTCGATACTTGCC
ATTGAAATAGAGAAGCAACGGCTTTCTGAGGATAATATGGAAGGAGAGAGAACCGACTCAACATCTC
CAGAATATTCAACTGCACTAGTAGGTAATGGGCAACCCAATGGCTGTAATAACGACTTCGTGGAGCTC
GATGATATTTCAATATCTTCGATTTCTGTATCTCTCGTACCCTTTTACCGTGGGACCCAAAAAATACAA
ATATTGCATCGAAATCTACCGTTGCAAGTTCAATGTACATGCCTGAAGATTCGATTTGGGATCAGTACG
AAATTTGTTGATCATGCTGGTCGGCCACGGTTGAATTTTGTGGTGGATGCATCCCCGAGTCTCTGCGAA
GTTCTTGACACAGCCGATAGTCTTGCCCAGAAGCTGTCAAGTGATTCTGGTAGCTCTTCTGAGTGGCGA
CCTGTGGTGACCAGGAAGAGAGGCTTCTTCAACTCTCCGACTATCCGGTTGCACCTACCGACTATAGC
AGATGGTGAACATACCCGATGCTCAACCGAGATATACCAGAAAGATAACTCGGCAACACAAAGGCTT
GTGTTTAGTAGATTCGACGCTAATGAGCTGGATTCCTTAATCACTCCCGGGAATATTGTGGATGTGAGT
TTCTCCTTGGACGTCTATGATTATCAGCAAAATGCCGGCATCCGATTGGTGGCAAAAAAACTGACTGTT
CATTCTACCTGATGGAATAACTGACCTTAAATTATTGTTTTTTATTTTCTTTTTTTACTATGTGAATGCTC
ATCCTGAGCGAGACAAGTTAATTACTGAAGGTTTATGTTGGATTTTTTTAAGAAAACTTGTAGTGGTTC
TTGGTTGTATTGTAAGGATGGTTTGATTTAACTTTGGTGCTGTTA
>PaNEN4
ACAGATGTAGTGTATGCTAGGATTACCCTTTAACAAGGACTTAGCTGAGCACACCCTTTGTCCAAACA
AAATATAACTTGAATTACAAGACAATCAATCTCCAATGCATGTCAAAAGAATATTCTTATGCCAAATT
CTAAATCTTGCTTTCAAATCGTCCCTCTCTCCGTTCATCTATAAATTCCAACAGCAATTAACAAAATCA
TCTAGCACCACAAAATTTAAAAAAAAAAAAGAATTATATAACCGTAAAAAATGGCTATTCCGGATAAA
ACCTATAAAAATATCATATCAGAAATTGTGTTCTTCGATTTAGAAACTACGTTTCCTAGCAAAACCGGA
CAAAAATTTTACATCCTGGAATTTGGCGCCATTGTCGTTTGTCCGAAAAAATTAATCGAGCTTGAGAGC
TATTGCACGCTGATTAGGCCACGGGATATATCGGTTGTGCCCGTCAAATCCGGACGGTCAGATGGAAT
AACGCGTGGGGCTATAGCCAATGCTCCTTGGTTTGATGAAGTTGCTGACAAGATTTTCGACGTACTTGA
TGGAAGAATATGGGCTGGCCACAATATTCAACGGTTCGACTGTGTTCGGATTAAAGAAGCATTTGAGC
AGATTAATCGGGCACCACCGAAGCCAGCTGGTATCATTGATTCATTAGGAGTGTTAACTGAGAAGTTT
GGAAGGAGGGCTGGAAATATGAAGATGGCAACACTGGCGGATTATTTTGGTCTTGGCCAGCAAAAGC
ATAGGAGCATGGAGGATGTAAGAATGAACTTGGAAGTCTTAAAGCATTGTGCAACTGTACTCCTTCTG
GAATCAAGCCTGCCCCCAGACGCATTAAATAGGCAATGCCGCAGCGGCCCAAGTGTTACTACAAGAA
GCAGAAGCTCACTGCAATCTACTAGTCCTGATAGTTTGAGTAACAAACCTTCAAGAATCAGAGGGAAA
ACAAATAGCAGGGAAGAGAGAAGCCGAAAATGGCCTCCATCATCAACTTCTCATCAGAGGGCTGTGC
CTTATGCAAGACAAAGTATTGGAAAGATGACAGATAGAGTGAAGAATATATTTTCCTACGCCAAGAGT
CGGCCTCTGAGTAACTTTTTGAAGCATTCGAAATCCCTAATTGGGTGAGAAAGAATAGGAAGAACTCA
TTCTTGCTAACAACAAAATTTGAAGCAGAAAAAAAAAAGTTGCCAGAGGTACATAATCAAGGCTTTGTC
ATCCTGTAGATGCAACCTTGTACTTGGTGAATACAGTATGTTACAACTGACAATGTCAACATGCAAATT
TGTTTAAGAGTTCAAACTTTGTAAGATGGTGTCGTTAT
>PaNAC45
ATTTCAAAAAAGGACCAGGAGACTGGTATATTTTACATATGGCACCGGTTTCATTGCCTCCCGGTTTCC
GATTTCACCCCACCGATGAAGAGTTAGTGGCTTATTACCTAAACAGAAAGATCAATGGCCGAAAAATC
GATCTTGAGGTCATCCAAGAAGTTGATCTCTACAAGTGTGAGCCATGGGACCTTCCAGGAAAGTCATT
ATTGCCAAGCAAAGATCTTGAATGGTACTTTTTTAGCCCTCGGGACCGCAAGTATCCCAACGGGTCAA
GGACGAACCGGGCAACAAAAGCCGGTTATTGGAAGGCCACAGGAAAGGACCGAAAAGTGAGTTCACA

AATGAGGGCAGTAGGGATGAAGAAGACCCTAGTTTATTACAGAGGGAGAGCGCCACATGGAGCTCGG
ACCGATTGGGTTATGCATGAATATCGTTTAGACGAAAGGGAATGTGATGCTCCCACTGGCTTGCAGGA
TGCTTATGCCCTTTGCCGTATATTCAAGAAAAGTTTGAACATCCCGAAAATCGGAGATCATTATGTTGC
TTCAGCCAGCGATCGGTCCTCCAGCATCGGCCTACATTGTGATGCAAAATATGAGAATAATGATCTAA
TTGATAGTTCTTCTGAATATCCAATGCCAATGAACT
